# Multidrug Resistance of *Gallibacterium anatis* Biovar Haemolytica Isolated from the Reproductive Tracts of Laying Hens

**DOI:** 10.3390/pathogens13110989

**Published:** 2024-11-12

**Authors:** Olimpia Kursa

**Affiliations:** Department of Poultry Diseases, National Veterinary Research Institute, 24-100 Puławy, Poland; olimpia.kursa@piwet.pulawy.pl

**Keywords:** *Gallibacterium anatis*, biovar haemolytica, multidrug resistance, oviduct, laying hens

## Abstract

Antimicrobial resistance is recognized worldwide as one of the greatest threats to human and animal health and the environment. To evaluate the resistance rate of *Gallibacterium anatis* biovar haemolytica, which contributes to bacteremia, oophoritis, ovarian follicle degeneration, salpingitis, decreased egg production, and increased mortality in hens, strains isolated from the reproductive tracts of layers were analyzed. The oviducts were taken from three hens from each of 10 flocks manifesting clinical signs related to laying. Twenty-two isolates of *G. anatis* biovar haemolytica collected from the three parts of the reproductive system were identified using MALDI-TOF and molecular methods. The biovar’s resistance to 19 antimicrobial substances was assessed using the disk diffusion (n = 8) and broth microdilution (n = 11) methods. The presence of virulence (*gtxA*, *gyrB*, and *flfA*) and antibiotic resistance (*bla_ROB_*, *aphA*, *tetB*, and *tetH*) genes was examined using PCR. All the isolates were resistant to four or more classes of antibiotics and were considered multidrug-resistant. All such isolates were resistant to tilmicosin, tylosin, and enrofloxacin, 88.2% were to tetracycline, and 82.4% to vancomycin. The *gtxA*, *gyrB*, *tetB*, and *tetH* genes were demonstrated. Considering the present prevalence of multidrug resistance among *G. anatis* biovar haemolytica isolates from laying hen reproductive tracts, surveillance in reproductive flocks is warranted.

## 1. Introduction

Multidrug resistance in commensal bacteria is an important problem worldwide. Multidrug resistance (MDR) in bacteria makes infections more difficult to treat, increasing treatment costs and leading to additional morbidity and mortality. This resistance is the result of evolution in commensal bacteria that encounter a variety of antibiotics prescribed to a host population treated multiple times with them [1]. In many cases, resistance is spontaneous and occurs independently of mutations or the acquisition of exogenous resistance determinants [1,2,3,4].

Gram-negative bacteria are more resistant to antimicrobials because of the presence of an outer membrane barrier to permeability. Phenotypically, the Gram-negative bacterium *G. anatis* has been divided into two different biovars based on their hemolytic properties: “haemolytica”, which causes β-hemolysis, and “anatis” as a non-hemolytic variant [5,6,7]. The haemolytica biovar of *G. anatis* has the greatest impact on poultry health, being associated with diseases of the systems in which it is found [8,9]. Under normal conditions, infection with *G. anatis* typically does not result in specific clinical symptoms or severe changes. However, in immunocompromised birds, *G. anatis* biovar haemolytica strains can enter the bloodstream, leading to septicemia and multiorgan inflammation [7,10]. The infection may extend beyond the upper respiratory tract, causing pericarditis, perihepatitis, liver necrosis, peritonitis, and enteritis [7]. In the reproductive system, it can manifest as oophoritis, hemorrhaging and rupture of ovarian follicles, follicular atresia, and salpingitis. In roosters, infections lead to reduced semen quality, lower sperm density, impaired motility, and loss of membrane integrity [7,8,11,12] *Gallibacterium anatis* primarily affects intensively farmed poultry and results in production losses, high mortality in broilers, and reduced egg production in laying hens. Recently, declines in egg-laying efficiency have been increasingly linked to acute or chronic salpingitis and reproductive tract inflammation caused by *G. anatis* [5,12,13,14,15].

These bacteria use adhesion and invasion as initial virulence mechanisms. After entering the bird’s body, they adhere to the epithelial cells of the oropharyngeal cavity or oviducts of hens [16]. During the disease process, the bacteria multiply rapidly and the synthesis of virulence factors begins [7,17,18]. Highly virulent strains of *G. anatis* adhere better to the primary oviduct epithelial cells of hens and trigger more intensive production of inflammatory cytokines (IL-6, TNF-α, and IFN-γ), indicating the induction of inflammation and the ability to cause damage to infected tissues [19]. Many specific virulence factors produced by G. anatis have been identified which can significantly affect the pathogenicity of a strain. These factors include the GtxA toxin; outer membrane vesicles; fimbriae; metalloproteases; biofilm; elongation factor thermo-unstable (EF-Tu) protein; clustered, regularly interspaced, short palindromic repeats (CRISPR); and integrative and conjugative elements (ICEs), whose presence significantly affects the development of the disease [5,7,17,18,19,20,21,22,23].

The most common route of transmission of this pathogen is horizontal through direct contact between infected birds. Another possibility for the spread of infection is the vertical route, through the ovary or fallopian tube or by the penetration of the pathogen through the eggshell, which significantly affects the developing embryo. In the case of infection in roosters, venereal transmission is also possible [5,7,8].

The spread of antibiotic-resistant bacteria in poultry with phenotypes extending to more than one class of antibiotics is a serious public health problem, and understanding the causes of MDR bacterial prevalence is important for reducing the incidence of resistance. Infections caused by *G. anatis* biovar haemolytica can lead to significant economic losses in the poultry industry. Understanding the action of this biovar in the avian reproductive system is crucial for the proper management and prevention of infections to ensure the health and productivity of poultry flocks and reduce the use of antibiotics in poultry production. In this work, we report on the multidrug resistance of *G. anatis* biovar haemolytica isolated from different parts of the reproductive tract of laying hens with signs of infection.

## 2. Materials and Methods

### 2.1. Sample Collection

From May 2022 to April 2024, diseased layer hens were submitted to the Department of Poultry Diseases at the National Veterinary Research Institute in Poland for routine diagnostic testing. In this study, birds were examined from 10 flocks from different parts of the country in which clinical signs associated with a decline in laying and egg quality problems had been found. After euthanizing three birds per flock by decapitation, necropsies were conducted under aseptic conditions, during which tissue and swab samples of the oviduct were collected. Reproductive tract samples comprising the ovarium, infundibulum, and uterus (as the middle sections of each part) were collected.

### 2.2. Isolation and Identification of Gallibacterium anatis

All oviduct samples were suspended in tris buffer (10 mM, pH 8.5; Eurx, Gdańsk, Poland) and homogenized using a manual LabGEN 125 device (Cole-Parmer, Vernon Hills, IL, USA). Every part of the homogenized tissue was transferred onto Columbia agar plates with 5% sheep’s blood and incubated at 37 °C under a 5% CO_2_ atmosphere for 24 h. The reference strain *Gallibacterium anatis* ATCC 43329 was used as a positive control. The bacterial colonies that produced β-hemolysis from the agar plate were transferred to a MALDI-TOF MS target plate and mixed with formic acid and α-cyano-4-hydroxycinnamic acid matrix solution. All mass spectra were analyzed with Bruker Daltonics v.4.1.70 software (Bruker Corporation, Billerica, MA, USA) and had index values at levels of 2.000–2.299.

### 2.3. Antibiotic and Multidrug Resistance

The disk diffusion and microbroth dilution methods were used to determine the antibiotic resistance of *G. anatis* biovar haemolytica isolates. The susceptibility of the isolates to 19 different antibiotics from 12 classes was tested. Resistance to 8 antibiotics was tested with the disk diffusion method (Oxoid, Basingstoke, UK), and resistance to 11 others was evaluated with the microbroth dilution method. The test applied a bacteria volume of 100 μL of 1.5 × 10^7^ CFU/mL (0.5 McFarland scale) distributed uniformly onto the Columbia agar with 5% sheep’s blood. Eleven antibiotics from nine classes were used: the quinolone florfenicol (30 μg), the β-lactams doxycycline (30 μg) and amoxicillin (25 μg), the fluoroquinolone enrofloxacin (5 μg), the polymyxin colistin (50 μg), the cephalosporin ceftazidime (30 μg), and the macrolides tilmicosin (15 μg) and tylosin (30 μg). The inhibition zones were interpreted visually.

Determination of minimum inhibitory concentration (MIC) was performed according to the Clinical and Laboratory Standards Institute M31-A2 standard [24] using a commercially prepared dehydrated panel for *Enterobacteriaceae* (Sensititre EU Surveillance Enterococcus EUVENC AST plate; Thermo Fisher Scientific, Waltham, MA, USA). The plates were incubated for 20–24 h at 37 ± 1 °C under aerobic conditions. Antimicrobial resistance (AMR) testing of *G. anatis* biovar haemolytica was performed from a fresh culture on agar and a suspension prepared at 0.5 McFarland density in 0.9% NaCl (bioMérieux, Marcy-l’Étoile, France), of which 10 μL was transferred to 11 mL of Mueller–Hinton broth (Thermo Fisher Scientific). The suspension was thoroughly vortexed and then 50 μL of it was added to each well of a plate. The plates contained different concentrations of 11 antibiotics: 8–1024 mg/L preparations of the aminoglycoside gentamicin, 0.5–64 mg/L concentrations of the β-lactam ampicillin, 0.12–16 mg/L solutions of the fluoroquinolone ciprofloxacin, respective 0.5–64 mg/L and 1–128 mg/L concentrations of the glycopeptides teicoplanin and vancomycin, 0.25–32 mg/L solutions of the lipopeptide daptomycin, 1–128 mg/L concentrations of the macrolide erythromycin, 0.5–64 mg/L preparations of the oxazolidinone linezolid, 0.5–64 mg/L solutions of the streptogramin quinupristin/dalfopristin, and respective 0.03–4 mg/L and 1–128 mg/L concentrations of the tetracyclines tigecycline and tetracycline. The plates were incubated for 24 h at 37 ± 1 °C. The MIC was defined as the lowest concentration preventing growth which was visible using a plate reader (Sensititre-TREK Vizion Digital MIC Viewing System; Thermo Fisher Scientific). Strains resistant to at least three classes of antimicrobials were identified as MDR.

### 2.4. DNA Extraction

Identified colonies of *G. anatis* biovar haemolytica were suspended in tris-EDTA buffer (10 mM, pH 8.5; Eurx, Gdańsk, Poland). Genomic DNA was extracted using a Maxwell RSC Cultured Cells DNA Kit (Promega, Madison, WI, USA) according to the manufacturer’s recommendations. The quantity and quality of the DNA was determined using the NanoDrop 1000 system (Thermo Scientific, Waltham, MA, USA). Extraction of DNA from the tris-EDTA used for sample preparation was conducted as a negative control. Samples were frozen at −20 °C until future analysis.

### 2.5. Real-Time PCR and PCR

The isolates of *G. anatis* were identified by real-time PCR using primers complementary to the *gyrB* gene described by Wang et al. [25], with slight modifications which were reported previously [26].

The PCR was conducted using specific *G. anatis* primers which amplified the 16S–23S rRNA gene [27] and with the temperature and time conditions described previously [28].

### 2.6. Virulence and Resistance Genes

The isolated DNA was used to perform a PCR test for the detection of the *gyrB*, *gtxA*, and *flfA* virulence genes. All samples were also tested for the presence of antibiotic resistance genes, namely *bla*_ROB_ (a β-lactam resistance gene), *aphA* (an aminoglycoside resistance gene), and *tetB* and *tetH* (tetracycline resistance genes). The primers described earlier by Algammal et al. were used [23].

### 2.7. Presence of Other Bacterial Pathogens

Detection of other bacteria was undertaken in swab samples taken from the reproductive tracts of hens. The swab samples were suspended in tris-EDTA buffer. A 10 μL volume of supernatant was inoculated onto MacConkey agar, tryptic soy agar, bile esculin azide agar, and Columbia agar plates with 5% sheep’s blood (PIWet-PIB, Pulawy, Poland). The plate with MacConkey agar was incubated at 37 °C for 24 h. The plates with tryptic soy agar, bile esculin azide agar, and Columbia agar were incubated at 37 °C under a 5% CO_2_ atmosphere for 24 h. The obtained colonies were verified by MALDI-TOF MS.

The remainder of the suspension from the oviduct swabs was used for DNA extraction. Genomic DNA was extracted using a QIAamp DNA Mini kit (Qiagen, Hilden, Germany) according to the manufacturer’s recommendations. Samples were frozen at −20 °C until future analysis was undertaken.

The presence of DNA of *Mycoplasma synoviae* and *M. gallisepticum* was detected by PCR with primers complementary to the *vlhA* gene for *M. synoviae* [29,30] and to the *mgc2* gene for *M. gallisepticum* [30,31]. The presence of *Ornithobacterium rhinotracheale* was tested in a real-time PCR targeting the 16S rRNA gene according to Abdelwhab et al. [32,33]. The PCR amplicons were separated by electrophoresis on a 2% agarose gel containing ethidium bromide and were visualized by ultraviolet transillumination.

### 2.8. Statistical Analysis

Statistical analysis was conducted using one-way ANOVA and the Mann–Whitney test to assess the significance of the presence of virulence genes and oviduct changes. A *p*-value of <0.05 was considered statistically significant. All analyses were performed using the Social Science Statistics program (www.socscistatistics.com, accessed on 13 October 2024).

## 3. Results

### 3.1. Isolation and Identification of Gallibacterium anatis Biovar Haemolytica

**Figure 1:**
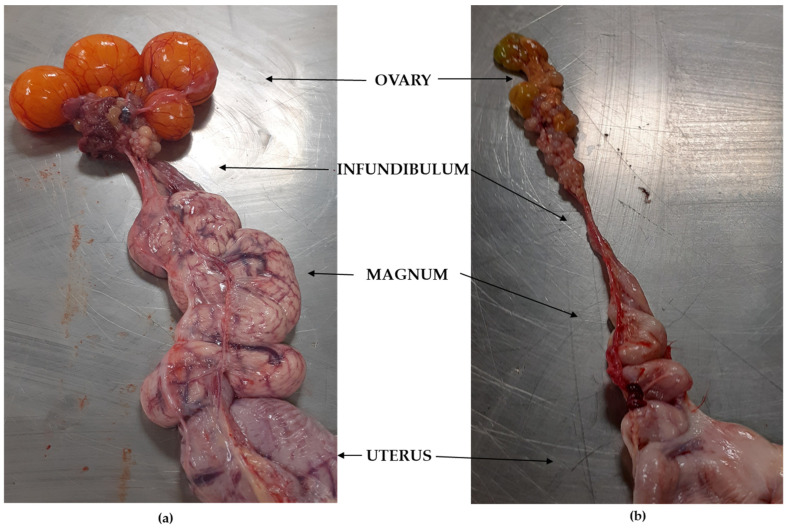
Ovarian and oviduct reproductive tract tissue of infected laying hens: (**a**) unchanged; (**b**) changed (degeneration of the ovarian follicles and reduced oviduct).

Post-mortem examination of laying hens most often found ovarian inflammation, degeneration of ovarian follicles, congestion or inactivity of the oviducts, or their non-functional state. Figure 1 shows the degeneration of the ovarian follicles and the reduction of the oviduct. Changes in the oviduct were found in 48.5% of the birds examined. There were birds in every flock in which at least one of the listed changes was found. Twenty-two isolates of *G. anatis* biovar haemolytica were obtained from the reproductive system organs of the laying hens, which were confirmed by MALDI-TOF MS. A summary of the obtained isolates’ origins is shown in Table 1. Seven isolates originated from the ovaries, fifteen from the uterus, but none from the infundibulum.

### 3.2. Antibiotic and Multidrug Resistance

**Figure 2:**
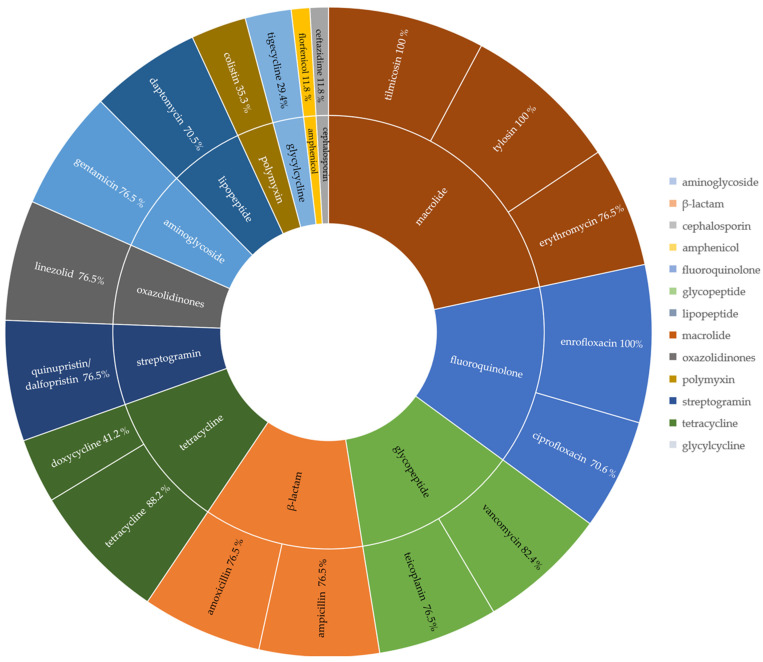
Antibiotics, their classes, and the percentages of strains which resisted them among *Gallibacterium anatis* biovar haemolytica isolates from layer hen reproductive tracts. Colors distinguish antibiotic classes.

The 22 isolates of *G. anatis* confirmed by MALDI-TOF MS were evaluated for antibiotic resistance. Seven isolates were obtained from the ovaries and fifteen from the uterus. Five isolates obtained from ovaries showed the same profile as isolates from the uterus and the other two showed a different profile. As a result, 17 distinct profiles were analyzed. Among the isolates, 100% were resistant to tilmicosin, tylosin, and enrofloxacin; 88.2% were to tetracycline; 82.4% to vancomycin; and 76.5% to erythromycin, linezolid, quinupristin/dalfopristin, ampicillin, amoxicillin, gentamicin, and teicoplanin. Ciprofloxacin and daptomycin were resisted by 70.6% of isolates and doxycycline by 41.2%. In contrast, 88.2% of strains were sensitive to florfenicol and ceftazidime, 70.6% were to tigecycline, and 64.7% to colistin (Figure 2).

**Figure 3:**
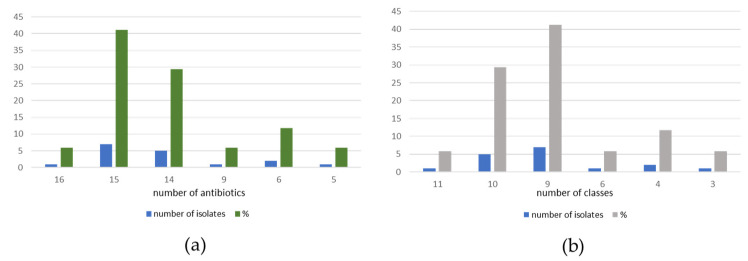
AMR of *G. anatis* biovar haemolytica isolates: (**a**) number and percentage of isolates resistant to different antibiotics. (**b**) number and percentage of isolates resistant to different classes of antibiotics.

This biovar of *G. anatis* as isolated from the reproductive tracts of layer hens presenting clinical cases exhibited 100% multidrug resistance. Most *G. anatis* biovar haemolytica isolates were resistant to 15 (41.2%) or 14 (29.4%) antibiotics belonging to 10 (29.4%) or 9 (41.2%) different classes. Among the isolates obtained, only one was resistant to 16 antibiotics (5.9%) belonging to 11 different classes. Two isolates (11.8%) showed resistance to six antibiotics from four different classes while single isolates showed resistance to nine (5.9%) and five (5.9%) antibiotics from six and three classes, respectively (Figure 2 and Figure 3).

Thirteen AMR profiles were found in the set of isolates analyzed. Unique AMR profiles were found in 10 isolates, and identical profiles were found in birds from the same flock (Table 2).

### 3.3. Real-Time PCR and PCR

DNA was extracted from the 22 isolates of MALDI-TOF MS–confirmed *G. anatis* biovar haemolytica. All samples in real-time PCR had a Ct range from 19.45 to 34.2. All isolates also showed two-band amplicons in PCR confirming the presence of *Gallibacterium* spp. The results of real-time PCRs and PCRs confirmed the presence of genetic material of *G. anatis* in all isolates.

### 3.4. Presence of Virulence and Resistance Genes

The *gtxA* and *gyrB* virulence genes were detected in 100% of the isolates, and the *flfA* virulence gene was detected in 22.7% of them. Statistically significant differences (*p* < 0.05) were found between the presence of virulence genes and changes in the oviducts of the hens. The *tetB* resistance gene was present in 100% of *G. anatis* biovar haemolytica isolates, while the *tetH* gene was found in 27.3% of them. The *aphA* and *bla_ROB_* genes were not detected in the examined isolates.

### 3.5. Presence of Other Bacterial Pathogens

In cultures from the oviducts of hens where *G. anatis* biovar haemolytica was found, *Staphylococcus hyicus*, *S. sciuri*, *S. chromogenes*, *S. simulans*, *S. aureus*, *Streptococcus alactolyticus*, *E. coli*, *Enterococcus cecorum*, *E. faecium*, and *Kurthia gibsonii* were identified by MALDI-TOF. The presence of *M. synoviae* DNA was found in oviducts of five laying hens. The isolated *M. synoviae* strains showed the greatest similarity to the MS-H (*Mycoplasma synoviae*-H) vaccine strain in phylogenetic analysis, which almost certainly originated from the vaccine given to the hens. However, genetic material of *O. rhinotracheale* was detected in six oviducts. No presence of *M. gallisepticum* DNA was observed in the tested samples.

## 4. Discussion

*Gallibacterium anatis* is an important pathogen found in poultry around the world. The symptoms occurring with biovar haemolytica infection can cause significant losses in poultry production. However, the biggest problem is the increasing multidrug resistance of this pathogen, which has been described by many scientists [6,14,26,34,35,36]. Several publications have described its resistance to a number of antimicrobial substances. The task of this study was to evaluate the multidrug resistance of isolates of *G. anatis* biovar haemolytica obtained from the reproductive tracts of hens showing clinical signs of infection.

Infections with *G. anatis* biovar haemolytica in laying hens frequently result in oviduct inflammation, swelling, and the accumulation of purulent secretions and fibrin deposits, along with degeneration of ovarian follicles. This leads to a reduction in egg production ranging from 3 to 18% under natural conditions due to damage to the oviducts and an overall decline in hen health [15,16,21,35,36]. In experimentally infected birds, egg production drops by as much as 66% and 47% during the first and third weeks post infection, respectively. Cumulative flock mortality rates between 0.06 and 4.9% have also been reported [12,19]. Samples from laying hens in this study showed lesions associated with ovarian inflammation, degeneration of ovarian follicles, and oviduct congestion or inactivity. *G. anatis* biovar haemolytica was isolated from oviduct samples, which may suggest their influence on the development of lesions as reported by other researchers [35,37,38,39].

The antibiotic resistance results of *G. anatis* isolates obtained from the reproductive systems of laying hens in several other studies suggested a high level of resistance to many antimicrobial agents commonly used in both veterinary and human medicine [8,23,35,39,40]. In studies conducted in the USA and Austria, high resistance to the macrolide antibiotic tylosin (85.3–100%) and tetracycline (88.2–100%) was observed, which is consistent with the results obtained in this study [34,35,41]. In studies conducted in Poland by Karwańska et al. [39], strains isolated from laying hens showed 50% resistance, which is less than that of isolates from the USA [41], Austria [35], and this study. Resistance to tetracyclines and macrolides seems to be common in various regions of the world, suggesting the limited effectiveness of these classes of antibiotics in treating infections caused by *Gallibacterium anatis*.

The varying susceptibility of the studied *G. anatis* strains to commonly used antibiotics nevertheless suggests that some treatment options remain effective, although their efficacy is not universal. In the study by Shabbir et al. [41], *G. anatis* isolates were resistant to penicillin (98%) but susceptible to cephalosporins (100%). However, among Austrian isolates, resistance to oxacillin (98.1%) and partial resistance to cefazolin (21.1%), ceftazidime (20.6%), and β-lactam antibiotics (up to 29%) was observed [35]. In this study, the isolates showed high resistance to ampicillin (76.5%) and amoxicillin (76.5%), but the isolates were susceptible to ceftazidime (88.2%). However, the resistance gene *bla*_ROB_ and *aphA* were not found in the tested isolates. Among isolates obtained from western Poland in earlier research, the isolates were fully resistant to penicillin (100%) and cefotaxime (100%) but fully susceptible to the cephalosporins ceftiofur and ceftazidime (100%) [39]. In studies conducted by Algammal et al. [23], resistance to penicillin and ampicillin was 95.9%. Resistance to both antibiotics in the fluoroquinolone class was high in the isolates obtained from hens in this study (100% were resistant to enrofloxacin and 70.6% to ciprofloxacin), which is consistent with the 91.2% obtained in the studies by Karwańska et al. Ref. [39] suggests that strains with high resistance to fluoroquinolones are present in Poland. However, in the strains isolated in Austria, resistance to enrofloxacin was lower at 58.2% [35], and was non-existent in strains in the USA, 100% of which were susceptible [41]. The growing resistance to several key antimicrobial agents further limits treatment options. Multidrug-resistant isolates also showed 76.5% resistance to erythromycin, linezolid, quinupristin/dalfopristin, ampicillin, amoxicillin, gentamicin, and teicoplanin. Resistance to daptomycin was 70.6% and resistance to doxycycline was 41.2%.

The high observed MDR in this study is comparable to the results observed in chickens in other countries, and not only in isolates from the reproductive system; isolates from the USA, Austria, Egypt, Germany, and Poland were resistant to many drugs, including enrofloxacin, macrolides, tetracyclines, and penicillin [5,8,23,35,41,42]. Studies conducted by Algammal et al. showing correlations between resistance to any antimicrobial agent and resistance to other specific agents suggest that resistance to one often coincides with resistance to others, which can further complicate treatment protocols for *G. anatis* infections [23].

The occurrence of MDR strains, particularly in the case of commonly used antibiotics, highlights the importance of careful management of antimicrobial agents in poultry farming. The antibiotics in use are becoming ever less effective against *G. anatis*, and alternative treatment methods or preventive measures are needed.

The most fully characterized virulence factor of *G. anatis* is the GtxA toxin, the protein responsible for the hemolytic activity of *G. anatis* biovar haemolytica. The GtxA protein is similar to the RTX toxins of the Pasteurellaceae family in terms of sequencing and to α-hemolysin (HlyA), which is found in *E. coli* [20,21,43]. The binding of the GtxA toxin to the actin present in host immune cells can alter cell structure and impede cell signal transduction, and thus the bacterium can avoid the host immune system [20,21,43]. Isolates tested in this study showed β-hemolysis, and the *gtxA* and *gyrB* virulence genes were confirmed in 100% of them. In contrast, the presence of the *flfA* virulence gene was found to a lesser extent, in 22.7% of the isolates. The harboring by *G. anatis* of a number of genes related to its ability to defend itself against antibiotics, such as those encoding the EF-Tu and CRISPR proteins, renders successful elimination of it from the bird’s body a major challenge. In addition to these genes, ICEs have been found in *G. anatis* strains, which contain antibiotic resistance genes and have the potential to pass them on to other bacteria [5,7,18,44].

The development of the disease caused by *G. anatis* is influenced by many physiological and environmental factors, which can affect the duration of infection and mortality of birds in the flock. An additional complicating or initiating factor in the development of symptoms may be exposure of the flock to mixed infections associated with bacterial or viral contamination. *Gallibacterium* can cause primary or secondary infections leading to more severe clinical symptoms. Because strongly developed clinical symptoms were present in the studied flocks, samples were tested for the presence of other pathogens that could escalate infections. In some flocks, mixed infections were found with *O. rhinotracheale*, *E. coli*, *M. synoviae*, *Enterococcus faecium*, *E. cecorum*, *Staphylococcus aureus*, or *Kurthia gibsonii.* The occurrence of mixed infections has already been described in different reports [15,45,46,47,48]. Some of these bacteria are unable to cause infection when they alone are the pathogen, but rather are opportunistic pathogens which cause significant losses in poultry production in co-infections.

## 5. Conclusions

In conclusion, the antimicrobial resistance of *G. anatis* biovar haemolytica indicates serious challenges in treating infections with this pathogen in poultry because high levels, of co-resistance and multi-resistance are observed in certain strains. The widespread resistance of *G. anatis* to a number of commonly used antibiotics and its possession of a number of genes associated with its defense and ability to transmit resistance to other bacteria call for a more cautious use of antimicrobials and the search for alternative treatment strategies.

## Figures and Tables

**Table 1:** Number of isolates by origin site in the layer hen reproductive tract.

Flock No.	Age of Birds (Week)	Ovary	Infundibulum	Uterus
1	30	1	0	1
2	28	1	0	2
3	31	0	0	1
4	31	1	0	2
5	28	0	0	1
6	28	1	0	1
7	30	2	0	2
8	27	1	0	2
9	29	0	0	1
10	28	0	0	2
	Total	7	0	15

**Table 2:** AMR profiles of the *G. anatis* biovar haemolytica isolates.

AMR Profile	Number of Isolates
AML, ENR, TY, TIL, AMP, CIP, DAP, E, CN, LZD, QD/SYN, TEC, TE, TGC, VA	2
AML, ENR, TY, CAZ, TIL, AMP, CIP, DAP, E, CN, LZD, QD/SYN, TEC, TE, TGC, VA	1
AML, ENR, TY, TIL, AMP, CIP, DAP, E, CN, LZD, QD/SYN, TEC, TE, VA	3
AML, FFC, ENR, TY, TIL, AMP, CIP, DAP, E, CN, LZD, QD/SYN, TEC, TE, VA	1
AML, ENR, TY, TIL, AMP, CIP, E, CN, LZD, QD/SYN, TEC, TE, TGC, VA	1
AML, ENR, TY, CT, TIL, AMP, CIP, DAP, E, CN, LZD, QD/SYN, TEC, TE, VA	1
ENR, TY, TIL, CIP, TE	1
DO, FFC, ENR, TY, CT, TIL	1
DO, ENR, TY, CT, TIL, QD/SYN, TEC, TE, VA	1
DO, ENR, TY, CT, CAZ, TIL	1
DO, AML, ENR, TY, CT, TIL, AMP, DAP, E, CN, LZD, TE, TGC, VA	1
DO, AML, ENR, TY, CT, TIL, AMP, DAP, E, CN, LZD, QD/SYN, TEC, TE, VA	1
DO, AML, ENR, TY, TIL, AMP, CIP, DAP, E, CN, LZD, QD/SYN, TEC, TE, VA	2
Total	17

AML—amoxicillin; ENR—enrofloxacin; TY—tylosin; TIL—tilmicosin; AMP—ampicillin; CIP—ciprofloxacin; DAP—daptomycin; E—erythromycin; CN—gentamycin; LZD—linezolid; QD/SYN—quinupristin/dalfopristin; TEC—teicoplanin; TE—tetracycline; TGC—tigecycline; VA—vancomycin; CAZ—ceftazidime; FFC—florfenicol; CT—colistin; DO—doxycycline.

## Data Availability

The data presented in this study are available on request from the corresponding author. The data are not publicly available because of legislation protecting privacy.
